# Ultraviolet light triggers the conversion of Cu^2+^-bound Aβ_42_ aggregates into cytotoxic species in a copper chelation-independent manner

**DOI:** 10.1038/srep13897

**Published:** 2015-09-09

**Authors:** Xiongwei Dong, Zhe Zhang, Dan Zhao, Yaojing Liu, Yan Meng, Yong Zhang, Dan Zhang, Changlin Liu

**Affiliations:** 1 Key Laboratory of Pesticide & Chemical Biology, Ministry of Education, and School of Chemistry, Central China Normal University, Wuhan 430079, Hubei; 2 School of Chemical and Materials Engineering, Hubei Polytechnic University, Huangshi, 435003 Hubei, China

## Abstract

Increasing evidence indicates that abnormal Cu^2+^ binding to Aβ peptides are responsible for the formation of soluble Aβ oligomers and ROS that play essential roles in AD pathogenesis. During studying the Cu^2+^-chelating treatment of Cu^2+^-bound Aβ_42_ aggregates, we found that UV light exposure pronouncedly enhances cytotoxicity of the chelator-treated and -untreated Cu^2+^-bound Aβ_42_ aggregates. This stimulated us to thoroughly investigate (1) either the chelation treatment or UV light exposure leads to the increased cytotoxicity of the aggregates, and (2) why the chelator-treated and -untreated Cu^2+^-bound Aβ_42_ aggregates exhibit the increased cytotoxicity following UV light exposure if the latter is the case. The data indicated that the controlled UV exposure induced the dissociation of Cu^2+^-free and -bound Aβ_42_ aggregates into SDS-stable soluble oligomers and the production of ROS including H_2_O_2_ in an UV light intensity- and time-dependent, but Cu^2+^ chelation-independent manner. Although we can’t fully understand the meaning of this finding at the current stage, the fact that the UV illuminated Aβ_42_ aggregates can efficiently kill HeLa cells implies that the aggregates after UV light exposure could be used to decrease the viability of skin cancer cells through skin administration.

Recently, the light regulation of protein or peptide aggregation started to attract attention. The catalytic photooxygenation was found to attenuate the aggregation and neurotoxicity of amyloid β (Aβ) peptides^1^. The aggregation and cytotoxicity of Aβ peptides were also shown to be inhibited by photodegradation in the presence of a designed fullerene derivative^2^,^3^. Moreover, the controlled ultraviolet (UV) exposure was observed to induce the native protein chicken egg white lysozyme to form fibrils under native conditions^4^. The main studies on the photocontrol of reversible amyloid formation were focused on the designed peptides modified with an azobenzene group or a photocaged analogue of lysine^5^,^6^,^7^,^8^. The trans to cis photoisomerization of the azobenzene group was shown to induce dissociation of the amyloids formed by different peptides^5^,^6^,^7^. The photocleavage of the photocaged analogue of lysine was observed to trigger disassembly of the amyloids formed by a modified Aβ_16–22_ mutant^8^. However, the light regulation of the aggregation and dissociation of metal-bound Aβ peptides remains to be explored.

The brains of Alzheimer’s disease (AD) patients are characterized by the deposition of amyloid plaques whose main component is Aβ peptides. Aβ_42_ is more neurotoxic and has a higher tendency to aggregate than Aβ_40_^9^. Remarkably high concentrations of Cu and Zn have been found within the amyloid deposits in AD-affected brains^10^. Increasing evidence indicates that the generation of soluble Aβ oligomers and reactive oxygen species (ROS) are two cytotoxic events that plays essential roles in the AD pathogenesis^11^,^12^,^13^,^14^,^15^,^16^,^17^,^18^. Binding of Cu^2+^ to Aβ peptides has been observed to be responsible for these two pathogenesis events^16^,^17^,^18^,^19^,^20^. Moreover, the Cu^2+^-dependent generation of ROS including H_2_O_2_ has been observed to occur during the Aβ oligomerization stages^21^. These observations inspire investigators to develop strategies to prevent Cu^2+^ from binding to Aβ peptides, to inhibit the generation of Aβ oligomers and ROS, and to promote the dissociation of Cu^2+^-bound Aβ aggregates through removal of Cu^2+^.

Removal of Cu^2+^ from Cu^2+^-Aβ complexes or aggregates through chelation is attracting extensive attention as a strategy to reduce the toxicity resulted from Cu^2+^-Aβ interactions. Two considerations underlie this strategy: the Cu^2+^-Aβ interactions should be reversible and binding of Cu^2+^ to Aβ peptides could be regulated by chelators. Thus, a variety of chelators against the abnormal Cu^2+^-Aβ interactions were designed and tested in *in vitro* and *in vivo* models^22^. The chelators clioquinol (CQ) and PBT2 (8-hydroxy quinoline analogs) showed for the first time the decreased formation of Aβ aggregates that resulted in improved cognition in clinical trials^23^,^24^,^25^. Subsequently, a large body of multifunctional chelators were prepared and examined *in vitro* as a potential reagent of the chelation treatment for AD^26^,^27^,^28^,^29^,^30^,^31^,^32^,^33^,^34^,^35^,^36^,^37^,^38^,^39^,^40^,^41^,^42^,^43^,^44^,^45^. Among them, a few of chelators, for example, CQ and its derivatives, were also reported to be capable of reducing Aβ peptide levels and ameliorating the Aβ toxicity by the restoration of metal homeostasis and of endocytic function^23^,^24^,^46^. Moreover, structures of the oligomers and intermediates during the formation and dissociation of Aβ aggregates were characterized by NMR under different conditions^47^,^48^,^49^,^50^,^51^.

Although the studies mentioned above indicate that the chelators can efficiently induce the dissociation of Cu^2+^-bound Aβ aggregates and inhibit ROS generation by targeting Cu^2+^, some reports suggest that this strategy may not reduce the toxicity resulted from the Cu^2+^-mediated Aβ aggregation^52^,^53^. Inspired by the fluorescent dye Thioflavin-T (ThT), the incorporation of a Cu^2+^-chelating moiety into the structural framework of this Aβ peptide-binding molecule produced several groups of bifunctional chelators to regulate Aβ aggregation^27^,^52^,^53^. They were found to result in the dissociation of Cu^2+^-bound Aβ aggregates into soluble oligomers, and suppress both the Cu^2+^-mediated formation of Aβ aggregates and ROS to a detectable extent. Because soluble Aβ oligomers were confirmed to be more toxic than amyloids^11^,^12^,^13^,^14^, this strategy targeting Cu^2+^, contrary to the expected, enhances toxicity of the Aβ aggregates^52^,^53^.

During studying the Cu^2+^-chelating treatment of Cu^2+^-bound Aβ_42_ aggregates, we unexpectedly found that the Cu^2+^-free and -bound aggregates exposed to UV light exhibited the increased cytotoxicity. Thus, in this study we examined the impact of the controlled UV exposure (≤400 nm) on the dissociation of the chelator-treated and -untreated Cu^2+^-bound aggregates. The results indicated that the controlled UV exposure triggered the dissociation of the aggregates into soluble oligomers, which was accompanied by the generation of ROS including H_2_O_2_, thereby leading to the notably increased cytotoxicity, irrespective of whether the aggregates were treated by the chelators (**FC-11** and **FC-11-1**) or not. It is noteworthy that Aβ_42_ aggregates after the controlled UV exposure can kill both neuron-like cells and cancer cells such as HeLa cells, suggesting that Aβ_42_ aggregates would has a potential use in the photochemical treatment of cancer, in particular, skin cancer.

## Results

### Synthesis and characterization

**Figure 1:**
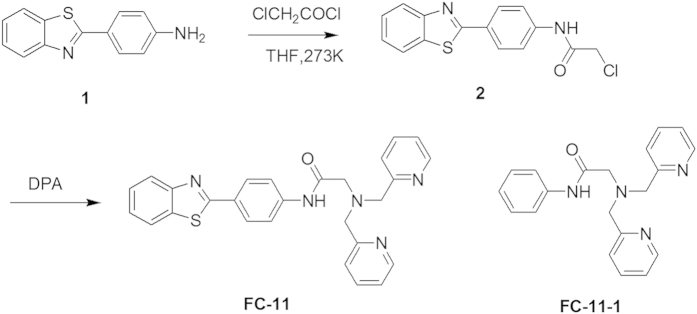
Synthesis of FC-11 and structure of FC-11-1.

The fluorescent chelator **FC-11** was designed by combining a Cu^2+^-chelating unit (DPA) and a ThT-based Aβ_42_ peptide-binding fluorescent unit^52^. The ThT-based fluorescent unit has a high affinity for protein amyloid fibrils^19^, and DPA has a moderate Cu^2+^-chelating ability (lg*K*_Cu_^2+^_-DPA_ = 9.3 M^−1^) relative to Aβ_42_ (lg*K*_Cu_^2+^_-Aβ42_ monomer = 5–10 M^−1^, lg*K*_Cu_^2+^_-Aβ42_ aggregate ~ 11 M^−1^, dependent on methods and conditions tested^16^,^20^). To enhance the Cu^2+^-chelating ability, the amide as a linkage group between the chelating and Aβ_42_ peptide-binding units was introduced into **FC-11**. **FC-11** was synthesized based on Fig. 1, and for comparison, **FC-11-1** that does not contain the Aβ_42_ peptide-binding fluorescent unit was prepared and characterized^54^.

Fluorescence measurements showed that the maximum emission of **FC-11** is at ~390 nm (excited at 330 nm), and is reduced with an increase in polarity of the organic solvents tested (Supplementary Fig. 1a). Moreover, this emission property of **FC-11** was not found to be changed with pH in pH 5.0–9.0 buffer or incubation time (for up to 48 h) in pH 7.4 buffer at 37 °C (Supplementary Fig. 1b). The divalent metal ions Cu^2+^, Co^2+^ and Ni^2+^ were observed to completely quench the fluorescence of **FC-11**, and the addition of Zn^2+^ or Cd^2+^ led to the significantly enhanced emission at given concentrations at pH 7.4 (Supplementary Fig. 1c,d). The fluorescence titration with a Cu^2+^ solution at pH 7.4 showed that **FC-11** forms a 1:1 complex with Cu^2+^ (Supplementary Fig. 2). These results suggested that the changes in the fluorescent property could be used to monitor Cu^2+^ chelation.

### Stability constants of Cu^2+^ complexes

Potentiometric titrations were carried out to determine the stability constant and solution speciation of Cu^2+^ complexes with **FC-11** and **FC-11-1**. The calculations performed using titration data (Supplementary Fig. 3a, b) showed that the Cu^2+^ complexes have large stability constants (log*K* = 17.78 for **FC-11** and 18.45 M^−1^ for **FC-11-1** at 25 °C), indicating that (1) the introduction of the amide linkage between the chelating and fluorescence units significantly increases the Cu^2+^-chelating ability of **FC-11** compared with that of its analogue (**FC-1**) and DPA^52^, (2) the fluorescence unit in **FC-11** has an impact on its Cu^2+^ chelation because **FC-11-1** has the stronger Cu^2+^ affinity than **FC-11**, and (3) the Cu^2+^ affinity of these chelators is much larger than that of Aβ_42_ peptides^16^,^20^. The high Cu^2+^ affinity of the chelating unit could underlie the Cu^2+^ sequestration of **FC-11** from Cu^2+^-bound Aβ_42_ aggregates under nearly physiological conditions.

Based on the stability constants, solution speciation diagrams were calculated for the coordination of the chelators with Cu^2+^ (Supplementary Fig. 3c, d). These diagrams suggested that 1:1 Cu^2+^-chelator complexes are the predominant species formed in the range of pH 4–8 for **FC-11** and pH 5–8 for **FC-11-1**. This pH range requisite for the optimal Cu^2+^ chelation completely covers the physiological pH range of formation of Cu^2+^-bound Aβ_42_ aggregates.

**Figure 2:**
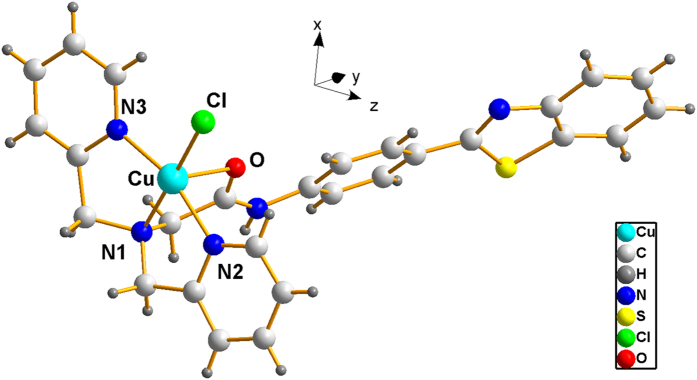
Structure of [Cu(FC-11)Cl]^+^. Solvent molecules and counteranions were omitted for clarity, all atoms were shown as sphere of arbitrary diameter.

In addition, the Cu^2+^ complex with **FC-11** was also characterized by X-ray crystallography (summary of data CCDC: **1043430**, and relevant crystal data listed in Supplementary Table 1). The structure revealed the formation of a 1:1 complex (Fig. 2), as indicated by the potentiometric and fluorescence titrations. The Cu^2+^ in this complex was coordinated to two pyridine N atoms, amine N atom and the amide O atom in **FC-11**, respectively, as expected.

### Sequestration of Cu^2+^

**FC-11** contains a 2-phenylbenzothiazole fragment that was designed by using the well-used fluorescent dye ThT for detection of the β-sheet structure of fibrillar Aβ aggregates. Binding of **FC-11** to the Aβ_42_ aggregates was examined by fluorescence measurements. Although the emission at 390 nm of **FC-11** (10 μM) was not observed to change in the presence of the Cu^2+^-free Aβ_42_ aggregates following incubation for 4 h, the fluorescence of **FC-11** was notably quenched by addition of the Cu^2+^-bound Aβ_42_ aggregates (10 μM, 1:1 for Cu^2+^/Aβ_42_) under the conditions tested, and the quenching extent was similar to that by the addition of 10 μM Cu^2+^ (Supplementary Fig. 4a). These results suggested that the presence of the Cu^2+^-free Aβ_42_ aggregates cannot significantly impact the fluorescence of **FC-11**, and the quenching of **FC-11** fluorescence does not provide direct support for its binding to the Cu^2+^-bound Aβ_42_ aggregates. To further explore interactions of the chelators with the aggregates, a ThT fluorescence competition assay was carried out by the addition of the chelators into the ThT-loaded aggregate solutions. A significant decrease in the ThT fluorescence intensity at 485 nm was found under the conditions tested regardless of the Cu^2+^-free or -bound aggregates (Supplementary Fig. 4b). A combination of these results indicated that the interactions with the chelators lead to the sequestration of Cu^2+^ from the Cu^2+^-bound Aβ_42_ aggregates.

The sequestration of Cu^2+^ from the Cu^2+^-bound Aβ_42_ aggregates (10 μM, 1:1 for Cu^2+^/Aβ_42_) was quantitatively examined by Cu^2+^ quenching of the **FC-11** (10 μM) fluorescence. The fluorescence at 390 nm of **FC-11** was found to be increasingly quenched upon addition of Cu^2+^, and this quenching had a linear relationship to Cu^2+^ concentrations (Supplementary Fig. 4c). Because the fluorescence of **FC-11** was impacted only by added Cu^2+^ (Supplementary Fig. 4a), its emission intensity at 390 nm following incubation for 4 h with the Cu^2+^-bound Aβ_42_ aggregates was used to evaluate the Cu^2+^ amount sequestered from the aggregates. Triple parallel experiments showed that the Cu^2+^ amount sequestered by 10 μM **FC-11** from the Cu^2+^-bound Aβ_42_ aggregates were 9.6, 12.1 and 9.7 μM (average, 10.5 μM), respectively, suggesting that **FC-11** forms a 1:1 complex via chelation of the Cu^2+^ ions bound to the Aβ_42_ peptides. Furthermore, the time courses indicated that the incubation period of 4 h is sufficient to allow **FC-11** to sequester the stoichiometric Cu^2+^ from the aggregates.

### Dissociation of Aβ_42_ aggregates

The results above obtained under visible light prompted us to examine dissociation of the Cu^2+^-free and -bound Aβ_42_ aggregates (10 μM Aβ_42_, Cu^2+^/Aβ_42_ = 1:1, 2:1) in darkness, and under UV or visible light, respectively, in the absence and presence of **FC-11** or **FC-11-1** at pH 7.4 and 37 °C. The incubation for 4 h was selected because the stoichiometric Cu^2+^ can be sequestered from the Cu^2+^-bound Aβ_42_ aggregates by the chelators. First, in the absence of chelators the dissociation of the Cu^2+^-free and -bound aggregates upon light exposure was monitored by SDS-PAGE. Both incubation in darkness and exposure to visible light for up to 24 h were not observed to lead to the appearance of Aβ_42_ oligomers. However, electrophoresis showed that when exposed to the increased intensity of UV light for 4 h the formation of Aβ_42_ oligomers was significantly increased, but the oligomer amounts did not have notable differences between the Cu^2+^-free and -bound aggregates (Supplementary Fig. 5a), indicating that the controlled UV exposure led to the dissociation of the aggregates into SDS-resistant oligomers, and the dissociation became more prominent as the UV light intensity rose. Moreover, a larger amount of Aβ_42_ oligomers were also observed for the 2:1 Cu^2+^-bound aggregates after exposure to 1500 Lux UV light for 4 h compared to that for the 1:1 aggregates (Supplementary Fig. 5b). Because the UV exposure at 1500 Lux can lead to the appearance of a large amount of Aβ_42_ oligomers, and corresponds to the strongest UV illumination in the midday sunlight, 1500 Lux UV light was selected for the following tests. In addition, the aggregates incubated in darkness, but not exposed to visible light, under the conditions tested were considered as control.

Then, the dissociation of the Cu^2+^-bound Aβ_42_ aggregates was examined in the presence of **FC-11** or **FC-11-1**. Fluorescence measurements indicated that **FC-11** can sequestrate the stoichiometric Cu^2+^ from the aggregates. We anticipated that the effective Cu^2+^ removal could promote the dissociation of the Cu^2+^-bound Aβ_42_ aggregates, as previously reported^10^,^23^,^24^,^25^,^26^,^27^,^28^,^29^,^30^,^31^,^32^,^33^,^34^,^35^,^36^,^37^,^38^,^39^,^52^,^53^. To obtain a line of support for this anticipation, the aggregates (Cu^2+^/Aβ_42_ = 1:1) were treated with 1 and 2 equivalent **FC-11** or **FC-11-1** of Aβ_42_ for 4 h, respectively, in darkness and under the conditions exposed to 1500 Lux UV light. Electrophoresis showed that the treatment with the chelators in darkness led to the formation of a few SDS-resistant Aβ_42_ oligomers as compared to the untreated samples (Supplementary Fig. 5c), indicating that the Cu^2+^ removal induces the dissociation of the aggregates to a observable extent, but the 2-fold addition of the chelators cannot notably improve the dissociation of the aggregates. Moreover, according to this qualitative result, it was difficult to find observable differences in the dissociation of the aggregates between two chelators. In contrast with this result, upon UV exposure a large amount of Aβ_42_ oligomers was found in the chelator-treated and -untreated aggregates. The binding of Cu^2+^ to Aβ_42_ peptides and Cu^2+^ removal from the aggregates both could slightly increase the formation of SDS-resistant Aβ_42_ oligomers (Supplementary Fig. 5c).

**Figure 3:**
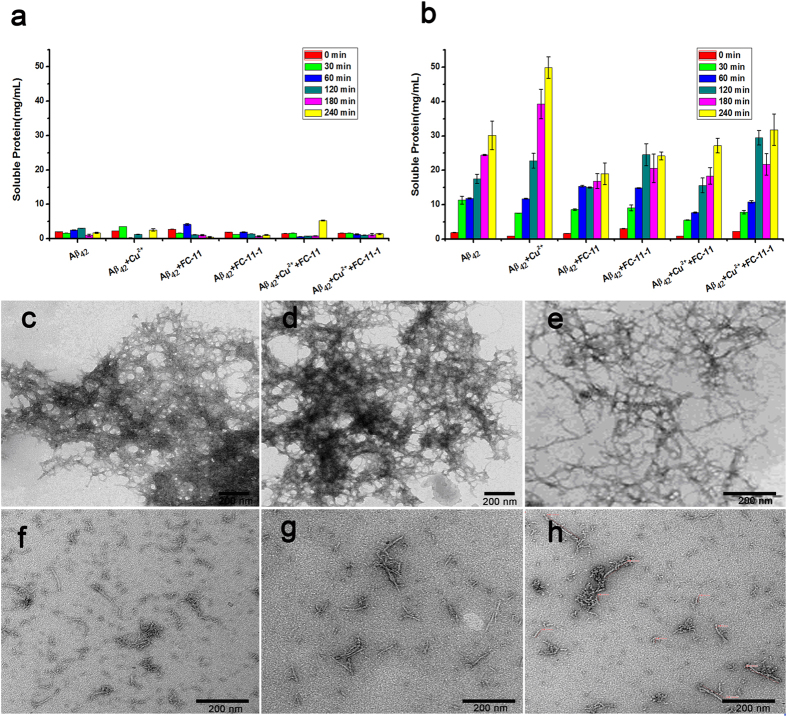
Analysis for dissociation of the Cu^2+^-free and -bound Aβ_42_ aggregates. The amount of soluble Aβ_42_ species in the supernatants provided by incubating 20 μM Cu^2+^-free and -bound Aβ_42_ (Cu^2+^/Aβ_42_ = 1:1) aggregates with 20 μM **FC-11** or **FC-11-1** in darkness (**a**) or under the conditions exposed to 1500 Lux UV light (**b**) for 0–4 h at pH 7.4 and 37 °C. (**e**–**h**) TEM imaging of Cu^2+^-free Aβ_42_ aggregates (**c**), Cu^2+^-bound Aβ_42_ aggregates (**d**), Cu^2+^-bound Aβ_42_ aggregates treated with **FC-11** for 4 h in darkness (**e**), Cu^2+^-free Aβ_42_ aggregates exposed to 1500 Lux UV light for 4 h (**f**), Cu^2+^-bound Aβ_42_ aggregates exposed to 1500 Lux UV light for 4 h (**g**), and Cu^2+^-bound Aβ_42_ aggregates after both **FC-11** treatment and 1500 Lux UV light exposure for 4 h (**h**).

To confirm the electrophoresis observations of the dissociation of the Cu^2+^-free and -bound aggregates, the soluble Aβ_42_ species in samples were evaluated by bicinchoninic acid (BCA) assays. The soluble species for BCA assays were produced by centrifugation following the chelator treatment of the Aβ_42_ aggregates for 0–4 h under the conditions incubated in darkness and exposed to 1500 Lux UV light, respectively. The soluble species in supernatants include Aβ_42_ monomers and oligomers. The data showed that the soluble Aβ_42_ species produced by the treatment in darkness are negligible, as occurred in control. Moreover, the amount of soluble Aβ_42_ species was not altered with the binding of Cu^2+^ to Aβ_42_ peptides, and the chelating treatment for 4 h could not significantly modify the amount of soluble Aβ_42_ species (Fig. 3a). However, the exposure to 1500 Lux UV light led to the formation of a large body of soluble Aβ_42_ species, and prolonging exposure periods further increased the formation of soluble Aβ_42_ species (Fig. 3b). It was noteworthy that this increased formation of soluble Aβ_42_ species was more prominent in the Cu^2+^-bound aggregates than in the Cu^2+^-free aggregates, suggesting that the binding of Cu^2+^ to Aβ_42_ peptides has an impact on the formation of soluble Aβ_42_ species. In addition, the sequestration of Cu^2+^ also had slight contribution to the increased formation of soluble Aβ_42_ species. These BCA assay data indicated that (1) the sequestration of Cu^2+^ in darkness does not results in the significant dissociation of the Cu^2+^-bound Aβ_42_ aggregates, (2) the controlled UV exposure outstandingly induces the dissociation of the aggregates into soluble Aβ_42_ species, and (3) the UV exposure provides the critical contribution to the increased dissociation of the aggregates in the presence of **FC-11** or **FC-11-1**. Obviously, these results were well consistent with the electrophoresis observations of the dissociation of the aggregates performed above.

To obtain more evidence to support the UV exposure-mediated dissociation of the Cu^2+^-free and -bound Aβ_42_ aggregates, the samples used in electrophoresis and BCA experiments were further examined under transmission electron microscope (TEM). The Aβ_42_ aggregates formed in the absence and presence of Cu^2+^ exhibit a well-organized fibrillar profile under TEM (Fig. 3c,d). Any notable alteration in the fibrillar profiles of the Cu^2+^-bound aggregates was not found for the treatment with the chelators in darkness (Fig. 3e). However, exposure to 1500 Lux UV light led to the disappearance of the well-organized fibrillar aggregates and the appearance of fibril-like aggregate fragments (Fig. 3f,g). A combination of the chelating treatment and controlled UV exposure also showed a profile of fibril-like aggregate fragments under TEM (Fig. 3h). Obviously, these TEM pictures indicated that the Cu^2+^ chelation in darkness does not cause the significant dissociation of the Cu^2+^-bound Aβ_42_ aggregates, and the UV exposure results in the conversion of the Cu^2+^-free and -bound aggregates into aggregate fragments (including soluble Aβ_42_ oligomers that are undetectable under TEM). Therefore, the TEM imaging verified the observations performed with both SDS-PAGE and BCA observations.

### Changes in secondary structures of Aβ_42_

**Figure 4:**
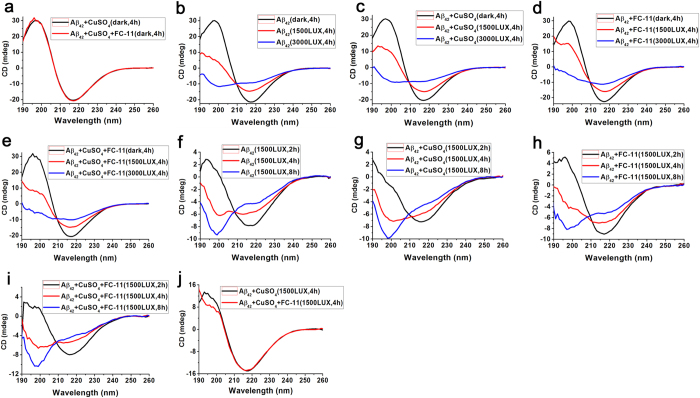
CD spectra of the Cu^2+^-free and -bound Aβ_42_ aggregates. (**a**) 10 μM Cu^2+^-bound Aβ_42_ aggregates (Cu^2+^/Aβ_42_ = 1:1) treated and untreated with 10 μM **FC-11** for 4 h in darkness. (**b**–**e**) 10 μM Cu^2+^-free (**b**,**d**) and -bound Aβ_42_ aggregates (**c**,**e**) incubated in darkness or exposed to 1500 and 3000 Lux UV light for 4 h in the absence (**b**,**c**) and presence of 10 μM **FC-11** (**d**,**e**). (**f**–**i**) 10 μM Cu^2+^-free (**f**,**h**) and -bound Aβ_42_ aggregates (**g**,**i**) exposed to 1500 Lux UV light for 2, 4 and 8 h in the absence (**f**,**g**) and presence of 10 μM **FC-11** (**h**,**i**). (**j**) 10 μM Cu^2+^-bound Aβ_42_ aggregates exposed to 1500 Lux UV light for 4 h in the absence and presence of 10 μM **FC-11.**

The results delineated above allowed us to anticipate that the controlled UV exposure-mediated dissociation of the Cu^2+^-free and -bound Aβ_42_ aggregates might be accompanied by changes in secondary structures of the aggregated Aβ_42_ peptides. To examine the conformational changes of Aβ_42_, the UV exposure-mediated dissociation of the aggregates suspended at pH 7.4 was monitored by CD spectra in the range of 190–260 nm, respectively, in the absence and presence of **FC-11** or **FC-11-1**. First, CD spectra were observed to be similar for the Cu^2+^-bound aggregates before and after treatment with the chelators in darkness (Fig. 4a), indicating that the secondary structure of Aβ_42_ is not significantly altered by the sequestration of Cu^2+^ in darkness, as expected. Then, the impact of UV exposure on the secondary structures of Aβ_42_ was observed. On the one hand, inspection of the CD spectra of four systems (Fig. 4b–e) found that compared to the CD spectrum in darkness, the positive peak at ~195 nm and the negative peak at ~216 nm, which are characteristic of a β-sheet-rich conformation, are significantly reduced, and even the positive peak is changed into a negative peak at ~200 nm that is characteristic of a random coil-rich conformation, as the UV light intensity was increased from 1500 to 3000 Lux. On the other hand, the CD spectra acquired at 1500 Lux UV light showed that the positive peak at 196 nm is changed into a strong negative peak, and the negative peak at ~216 nm is also markedly reduced with prolonging exposure from 2 to 8 h (Fig. 4f–i). Moreover, these alterations in the CD peaks were not found to be capable of correlating with the presence of **FC-11** or **FC-11-1**, but more distinct in the Cu^2+^-bound aggregates than in the Cu^2+^-free aggregates. The CD data indicated that the enhanced UV illumination and prolonged exposure both can convert the secondary structures of the Aβ_42_ peptides from β-structures to random coils in a Cu^2+^ sequestration-independent manner, and the binding of Cu^2+^ to Aβ_42_ makes these changes in secondary structures more notable. In addition, the comparison of CD spectra indicated that the Cu^2+^ chelation of **FC-11** can enhance the UV light-induced alteration in secondary structures of the aggregated peptides (Fig. 4j). Taken together with these CD results, the secondary structures of the Aβ_42_ peptides was found to exhibit an UV exposure intensity- and time-dependent, but Cu^2+^ sequestration-independent alteration during the UV light-mediated dissociation of the Cu^2+^-bound and -free aggregates.

### Generation of ROS

In order to further understand both the UV light-mediated dissociation of the Cu^2+^-bound and -free Aβ_42_ aggregates and the alterations in secondary structures of the Aβ_42_ peptides, electron paramagnetic resonance (EPR) was used to detect ROS generated by the aggregates incubated in darkness and exposed to the controlled UV exposure, respectively, in the absence and presence of **FC-11** or **FC-11-1**. Increasing evidence shows that H_2_O_2_ is one of the main ROS produced during Aβ aggregation in a Cu^2+^-dependent manner^10^,^16^,^20^,^21^. A Fenton reaction between H_2_O_2_ and added ferrous sulfate generates hydroxyl radicals (∙OH). ∙OH reacts immediately with DMSO contained in solutions to generate methyl radical (∙CH_3_)^55^,^56^. ∙CH_3_ reacts with the spin trap DMPO to form the adduct radical DMPO∙-CH_3_ that provides a typical 6-line EPR spectrum^56^,^57^. Therefore, the production of ROS such as H_2_O_2_ can be indicated by the spin trap EPR confirmation of the appearance of ∙CH_3_.

**Figure 5:**
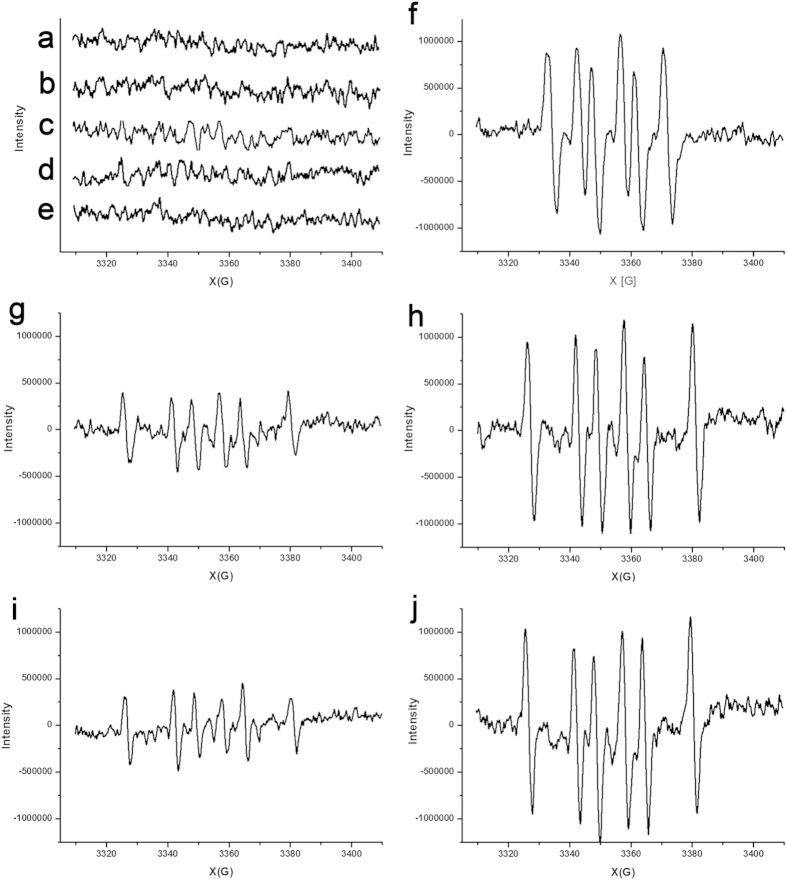
DMPO spin trap EPR detection of H_2_O_2_ generation. (**a**–**d**) Spin trap EPR spectra of pH 7.4 phosphate buffer containing 1% DMSO (**a**), 10 μM CuSO_4_ solution (**b**), 10 μM **FC-11** (**c**) and 10 μM **FC-11** complex solution of Cu^2+^ (**d**) after exposure to 1500 Lux UV light 4 h. (**e**,**f**) Spin trap EPR spectra of Cu^2+^-free aggregates in the presence of **FC-11** following incubation in darkness (**e**) or exposure to 1500 Lux UV light (**f**) for 4 h. (**g**,**h**) Spin trap EPR spectra of Cu^2+^-bound aggregates in the absence of **FC-11** following incubation in darkness (**g**) or exposure to 1500 Lux UV light (**h**) for 4 h. (**i**,**j**) Spin trap EPR spectra of Cu^2+^-bound aggregates in the presence of **FC-11** following incubation in darkness (**i**) or exposure to 1500 Lux UV light (**j**) for 4 h.

First, EPR measurements were carried out for controls under the conditions incubated in darkness or exposed to 1500 Lux UV light for 4 h by adding DMPO and FeSO_4_. In these EPR experiments, the 6-line ∙CH_3_ and 4-line ∙OH EPR spectra^56^ all were not found (Fig. 5a–d, Supplementary Fig. 6), indicating that there are not sources to generate H_2_O_2_ in the 1% DMSO-containing phosphate buffer, in the CuSO_4_ solution, or in the chelator solutions and their Cu^2+^ complex solutions under the conditions tested.

Then, EPR spectra were acquired for the Cu^2+^-free and -bound Aβ_42_ aggregates incubated in darkness and exposed to 1500 Lux UV light for 4 h in the presence and absence of **FC-11** or **FC-11-1**. The Cu^2+^-free aggregates all exhibited 6-line ∙CH_3_ EPR spectra following to UV exposure, whereas the aggregates in darkness did not exhibit similar behavior (Fig. 5e,f; Supplementary Fig. 6), regardless of whether the chelators were added or not. This result suggested that the controlled UV exposure induces the Cu^2+^-free aggregates to generate H_2_O_2_, but only the incubation in darkness does not. For the incubation of the Cu^2+^-bound aggregates without the chelators, the EPR signals observed for the samples incubated in darkness were much weaker than those for the samples exposed to UV light (Fig. 5g,h), indicating that the Cu^2+^-bound aggregates are a source of H_2_O_2_ generation, and the UV exposure causes this source to increase the generation of H_2_O_2_. Similarly, the incubation with the chelators allowed the Cu^2+^-bound aggregates exposed to UV light to increase the generation of ROS compared to those treated in darkness (Fig. 5i,j). In addition, the difference in EPR signal intensity between the **FC-11**-untreated and **-**treated Cu^2+^-bound aggregates in darkness was not observed to be pronounced (Fig. 5g,i), suggesting the sequestration of Cu^2+^ is not a efficient pathway to inhibit the generation of ROS. The strong EPR signal shown in Fig. 5f,h,j indicated that the UV light plays a critical role in the generation of ROS in the **FC-11**-treated and -untreated Cu^2+^-bound and Cu^2+^-free aggregates. Similar observations were performed for the aggregates treated with **FC-11-1** under the conditions tested (Supplementary Fig. 6). These EPR results revealed that (1) the controlled UV exposure enables both Cu^2+^-bound and Cu^2+^-free Aβ_42_ aggregates to become a robust source to generate ROS, whereas in darkness only the Cu^2+^-bound aggregates can generate H_2_O_2_; (2) the Cu^2+^-bound aggregates produce less H_2_O_2_ in darkness than in UV exposure; (3) the Cu^2+^ sequestration slightly inhibits the generation of ROS in the Cu^2+^-bound aggregates not only in darkness but also under UV light; (4) UV exposure provides a main contribution to the generation of ROS.

### Cytotoxicity

The above-mentioned results indicated the noticeably increased formation of soluble Aβ_42_ oligomers and H_2_O_2_ in the UV exposure-mediated dissociation of the chelator-treated and -untreated Cu^2+^-bound and Cu^2+^-free Aβ_42_ aggregates. Evidence reveals that the formation of soluble Aβ_42_ oligomers and ROS are critical cytotoxic events in AD pathogenesis^11^,^12^,^13^,^14^,^15^,^16^,^17^,^18^. Thus, it is necessary to estimate the changes in toxicity for the aggregates when incubated in darkness and exposed to UV light, respectively, in the absence and presence of **FC-11** or **FC-11-1**. To carry out this toxic estimation, the relative viability was measured by MTT on SH-SY5Y and HeLa cell lines, respectively.

**Figure 6:**
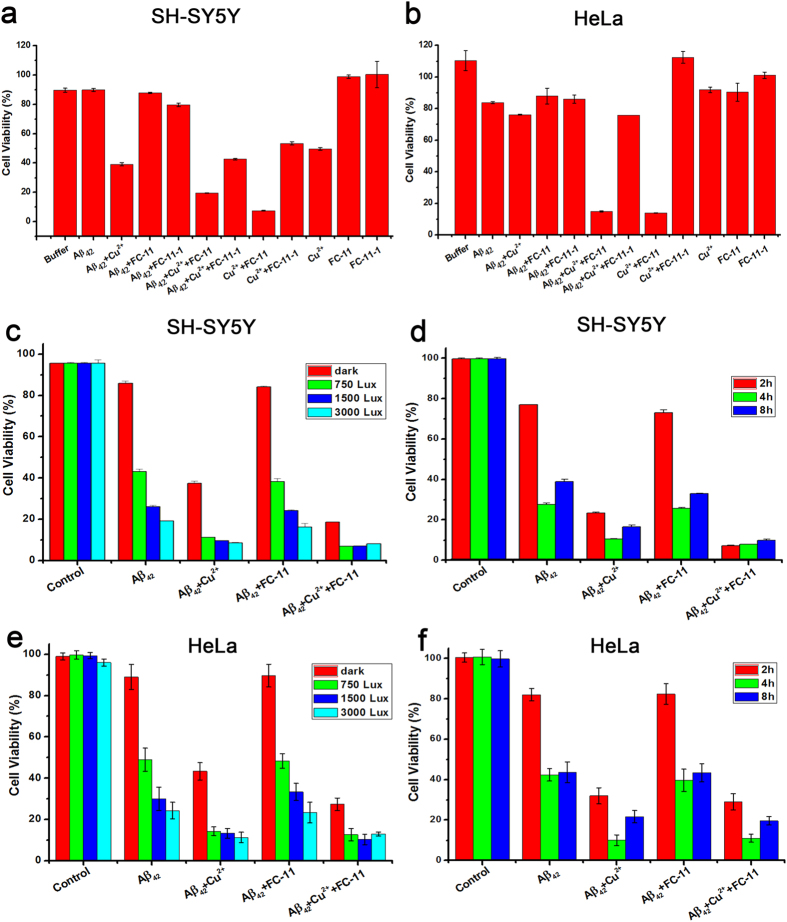
Estimation of toxicity on SH-SY5Y and HeLa cells. (**a**,**b**) SH-SY5Y (**a**) and HeLa cell (**b**) viability estimated by MTT for 10 μM cupric sulfate, 10 μM **FC-11** or **FC-11-1** and their Cu^2+^ complexes, and 10 μM Cu^2+^-bound and -free Aβ_42_ aggregates treated for 4 h, respectively, with and without 10 μM **FC-11** or **FC-11-1** in darkness. (**c**,**e**) SH-SY5Y (**c**) and HeLa cell (**e**) viability of 10 μM Cu^2+^-bound and -free Aβ_42_ aggregates exposed to 700, 1500 and 3000 Lux UV exposure for 4 h, respectively, in the presence and absence of 10 μM **FC-11**. (**d**,**f**) SH-SY5Y (**d**) and HeLa cell (**f**) viability of 10 μM Cu^2+^-bound and -free Aβ_42_ aggregates exposed to 1500 Lux UV exposure for 2, 4, and 8 h, respectively, in the presence and absence of 10 μM **FC-11**.

As contrast, the relative viability was estimated for the SH-SY5Y and HeLa cells exposed to the aggregates treated, respectively, with and without the chelators in darkness. The data showed that the viability of the SH-SY5Y cells, respectively, exposed to the chelators alone and to the chelator-treated and -untreated Cu^2+^-free aggregates was close to that of control (Fig. 6a), indicating that both the Cu^2+^-free aggregates and the chelators are low- and non-cytotoxic species in darkness. The viability of the cells exposed to the **FC-11**-treated Cu^2+^-bound aggregates or the Cu^2+^ complex of FC-11 was about half of that (~50%) of the untreated and **FC-11-1**-treated aggregates, or the Cu^2+^ complex with **FC-11-1**, or Cu^2+^ ions alone (Fig. 6a). The data demonstrated that (1) the strong cytotoxicity of the **FC-11**-treated Cu^2+^-bound aggregates, which is similar to that of the Cu^2+^ complex of **FC-11**, could be attributed to the formation of a **FC-11** complex via sequestering Cu^2+^ from the aggregates; (2) the **FC-11-1** complex with Cu^2+^ has less cytotoxicity than the **FC-11** complex, suggesting the incorporation of the Aβ_42_ aggregate-binding moiety can significantly increases the cytotoxicity of the **FC-11** complex likely through an indirect interaction between the moiety and the coordinated Cu^2+^; (3) the cytotoxicity resulted from the binding of Cu^2+^ to Aβ_42_ peptides is not significantly altered with the **FC-11-1** treatment. In addition, a comparison of the results obtained in darkness showed that the **FC-11**-treated Cu^2+^-bound aggregates and the **FC-11** complex with Cu^2+^ are also high toxic to the HeLa cells, and the Cu^2+^-bound aggregates are much higher toxic to the neuron-like cells than to HeLa cells (Fig. 6b).

For the cells incubated with the **FC-11**-treated Cu^2+^-bound and Cu^2+^-free Aβ_42_ aggregates exposed to the varied intensity and periods of UV exposure, the change in viability was estimated for SH-SY5Y cells. As the cytotoxicity was not significantly modified by Cu^2+^ chelation of **FC-11-1**, the **FC-11-1**-treated aggregates in darkness were ruled out of this estimation. First, the data showed that the viability is decreased with enhancing the UV exposure from 750 to 3000 Lux for the cells exposed to the Cu^2+^-free aggregates treated, respectively, with and without **FC-11**, whereas the cells exposed to the Cu^2+^-bound aggregates almost all die (viability ≤10%) irrespective of both UV light intensity and Cu^2+^ sequestration (Fig. 6c). Then, by fixing UV light at 1500 Lux, exposure for 4 h dramatically decreased viability relative to that for 2 h, but exposure for 8 h slightly increased the viability relative to that for 4 h for all the aggregates (Fig. 6d). In addition, the aggregates incubated in darkness were observed to have similar viability to that in controls (Fig. 6a). These results indicated that (1) the controlled UV exposure makes all the forms of the Aβ_42_ aggregates become toxic to SH-SY5Y cells, (2) the Cu^2+^-bound aggregates exposed to UV light can kill almost cells, and (3) **FC-11** chelation of Cu^2+^ enhances the cytotoxicity of the Cu^2+^-bound aggregates exposed to UV light.

The aggregates tested in the SH-SY5Y cytotoxic experiments were also estimated for the toxicity to HeLa cells (Fig. 6e,f). The similar results obtained indicated that the controlled UV exposure, instead of the sequestration of Cu^2+^, is a critical factor that causes the aggregates to become high toxic to HeLa cells. This result promotes us to propose that UV exposure might make the aggregates kill cancer cells including skin cancer cells.

## Discussion

We have reported that the finding that the treatment with an analogue of **FC-11**, **FC-1** that comprises an Aβ aggregate-binding fluorescent and a Cu^2+^-chelating unit, enhanced the cytotoxicity of the Cu^2+^-bound Aβ_40_ aggregates because of the dissociation of the treated aggregates into soluble Aβ oligomers^52^. Although this finding has been confirmed by a bifunctional chelator series^53^, we yet designed **FC-11** that has the stronger Cu^2+^-chelating ability from Cu^2+^-bound Aβ aggregates than **FC-1**. During absorption spectroscopic experiments, the Cu^2+^-bound Aβ_42_ aggregates treated with **FC-11** were found to have much larger cytotoxity than those untreated after undergoing UV-visible light exposure for given periods. To prove the anticipation that this enhanced cytotoxity should be attributed to the interaction between the fluorescent unit and the Cu^2+^ ion in the resulted chelate, **FC-11-1** was prepared by removing the fluorescent unit from **FC-11** according to the reported procedure^54^. The data showed that the **FC-11-1** complex with Cu^2+^ does have much less cytotoxity than the **FC-11** complex under the conditions tested (Fig. 6).

We noticed two facts that the **FC-11**-treated and -untreated Cu^2+^-free and -bound Aβ_42_ aggregates exhibit the UV exposure intensity- and time-dependent cytotoxicity, and the **FC-11** treatment considerably enhances the cytotoxity of Cu^2+^-bound Aβ_42_ aggregates in a UV exposure-independent manner (Fig. 6). These two facts indicated that the controlled UV exposure and **FC-11** chelation of Cu^2+^ both contribute to the enhanced toxicity of the Cu^2+^-bound Aβ_42_ aggregates, but only UV exposure leads to the noticeably enhanced toxicity. The critical evidence was not found to support a cooperative effect in the enhanced toxicity of the Cu^2+^-bound Aβ_42_ aggregates between UV exposure and **FC-11** chelation of Cu^2+^ , as the toxicity enhanced by these two processes was accumulative.

Accumulated evidence has proved neurotoxicity of soluble Aβ_42_ oligomers and H_2_O_2_^11^,^12^,^13^,^14^,^15^,^16^,^17^,^18^. Here, the formation of soluble Aβ_42_ oligomers and of H_2_O_2_ was indicated, respectively, with SDS-PAGE, BCA protein assay, TEM imaging (Fig. 3) and spin trap EPR (Fig. 5) during the UV exposure-mediated dissociation of the chelator-treated and -untreated Cu^2+^-free and -bound Aβ_42_ aggregates. An explanation could be proposed for the generation of ROS. UV photochemical oxidation of the tyrosine (Tyr) including the Tyr residues completely buried in the structurally well-defined proteins has been reported to occur via a proton-coupled electron transfer (PCET) process, yielding a significant amount of the radical Tyr-O∙ that slowly decays via an intermolecular radical-radical dimerization reaction^58^,^59^. The dioxygen molecules in solutions could act as a proton and electron acceptor in the PCET process to yield H_2_O_2_. This report, together with our observation that the freshly prepared Aβ_42_ sample exposed to UV light did not produce both H_2_O_2_ and oligomers, allow us to hypothesize that the Tyr residues in the aggregated Aβ_42_ peptides could form a dityrosine through UV photochemical oxidation because of their close proximity to each other in the aggregates. This suggests that the UV-photooxidation of the Tyr residues might lead to interchain-crosslinking of the aggregated Aβ_42_ peptides because each Aβ_42_ peptide contains only one Tyr residue. The resulted electrons and protons (or hydrogen atoms) might react with O_2_ in solutions to generate ROS including H_2_O_2_. In addition, the ozone provided by UV exposure might also contribute to the generation of ROS, because the oxygen atom yielded by the decomposition of the ozone also is an electron and proton acceptor. Obviously, to explain how UV exposure makes the aggregates become into a source of these neurotoxic species needs us to conduct thorough investigations.

The hypothesis mentioned above could be used to explain the UV exposure-mediated dissociation of the Cu^2+^-free and -bound Aβ_42_ aggregates. The interchain-crosslinking through the formation of dityrosines may bring about the conformational conversion of the aggregated Aβ_42_ peptides from β-sheets to random coils, as demonstrated by the CD data (Fig. 4). Moreover, the methionine residues in the aggregated peptides may be oxidized by H_2_O_2_^60^, also likely leading to the secondary structural alteration of β-sheets to random coils. The oxidation of the Aβ_42_ peptides may not exhaust H_2_O_2_ generated during UV exposure. The secondary structural alteration and oxidation of the Aβ_42_ peptides can make the aggregates dissociate into soluble oligomers. The soluble oligomers stable in 2% SDS with boiling can be attributed to formation of the covalent-crosslinked peptides. The finding that endogenous Aβ oligomer samples were boiled in the sample buffer containing 1–2% SDS for SDS-PAGE experiments without disruption of the oligomers^61^,^62^ leads us to hypothesize that the endogenous oligomers might be covalently crosslinked in their cellular environments.

**Figure 7:**
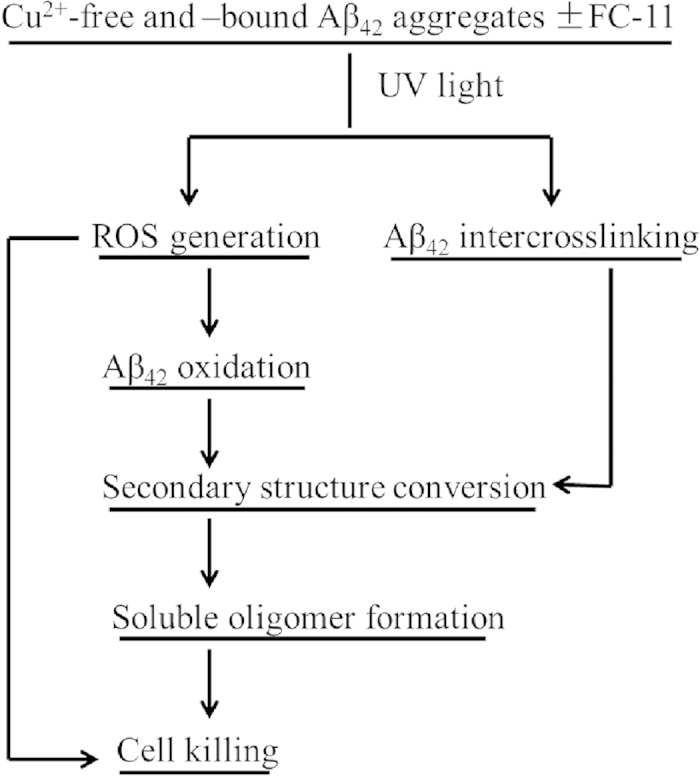
Proposed pivotal events in the UV exposure-mediated dissociation of the Cu^2+^-bound and -free Aβ_42_ aggregates.

By drawing together the results presented here with the discussion described above, several critical steps can be proposed in the controlled UV exposure-mediated dissociation of the Cu^2+^-free and -bound aggregates (Fig. 7). The UV exposure of the chelator-treated and -untreated aggregates results in the generation of H_2_O_2_ which might be accompanied by interchain-crosslinking of the aggregated Aβ_42_ peptides. Part of the H_2_O_2_ might be consumed for the oxidation of the aggregated Aβ_42_ peptides. The interchain-crosslinking and oxidation contribute to the secondary structural alterations that lead to the formation of soluble Aβ_42_ oligomers. Obviously, the neuron-like cells can be killed by both ROS and soluble Aβ_42_ oligomers.

In this study, we only described a photochemical phenomenon on the formation of toxic Aβ_42_ oligomers and ROS during the UV light-mediated dissociation of the Cu^2+^-bound aggregates in a Cu^2+^ chelation-independent manner. Obviously, we cannot understand significance of these results at least in the current stage because UV light cannot arrive at human brains. However, the fact that the incubation with the aggregates exposed to UV light resulted in less than 20% viability of HeLa cells (Fig. 6d,e) implicates that the UV illuminated aggregates can also kill cancer cells. It is possible that a photochemical treatment pathway of cancer is developed based on the finding delineated in this study. For example, as a potential use of the photochemical treatment, skin cancer might be treated by the Aβ_42_ aggregates loaded on skin and exposed to UV light. Photocontrolling the activity of biomolecules is providing new opportunities for the study of biological processes at the signal-cell level in a living organism^63^,^64^,^65^. Major efforts in photochemical control of cell activity can make us obtain more disease treatment-associated insights into this kind of study.

## Summary

In this study, **FC-11** designed by the introduction of an amide linkage group between the chelating and Aβ_42_ peptide-binding units has much stronger Cu^2+^-chelating ability than its analogue **FC-1**^52^. The chelator forms a 1:1 complex by sequestering the stoichiometric Cu^2+^ from the Cu^2+^-bound Aβ_42_ aggregates. However, this Cu^2+^ sequestration cannot efficiently promote the dissociation of the Cu^2+^-bound Aβ_42_ aggregates. The controlled UV exposure was observed to trigger the dissociation of the chelator-treated and -untreated Cu^2+^-bound aggregates into SDS-stable soluble oligomers in an UV exposure intensity- and time-dependent, but Cu^2+^ chelation-independent manner. The dissociation of the aggregates co-occurs with both the generation of ROS including H_2_O_2_ and the alterations in secondary structures of the aggregated peptides. Obviously, the formation of soluble Aβ_42_ oligomers and ROS contributes to the noticeably increased neurotoxicity of the aggregates. It is noteworthy that the UV illuminated Aβ_42_ aggregates can efficiently kill HeLa cancer cells, inspiring us to consider the Aβ_42_ aggregates exposed to UV light as a photochemical killer of cancer cells, e.g., as a killer of skin cancer cells through skin administration. Although here we only described a photochemical phenomenon regarding the formation of toxic Aβ_42_ oligomers and ROS during the UV exposure-mediated dissociation of the Cu^2+^-free and -bound aggregates, it is possible that the developments in photocontrolling the activity of biomolecules at the signal-cell level in a living organism might make us obtain more disease treatment-associated insights into this photochemical phenomenon.

## Methods

### General methods

All reagents were purchased from commercial sources (e.g., Sigma) and directly used unless stated otherwise. Solvents were purified by the most used methods. All solutions and buffers were prepared with using metal-free water that was passed through a Millipore-Q ultrapurification system. Elementary analysis was carried out on a Vario EL III elementary analysis instrument. UV-Vis spectra were recorded on an analytik jena Specord 210 spectrophotometer. ^1^H and ^13^C NMR spectra were recorded on a Varian Mercury 400 spectrometer at 400 and 100 MHz, respectively. Electrospray ionization mass spectra (ESI-MS) were acquired on an Applied Biosystems API 2000 LC/MS/MS system.

### Synthesis and X-ray structure of [Cu(FC-11)Cl]^+^

4-Benzothiazol-2-yl- benzenamine, **1**, was synthesized and characterized based on our reported procedure^52^. **1** (10 mmol) was dissolved in anhydrous THF (40 mL), and chloroacetyl chloride (11 mmol) was added into the THF solution in an ice-water bath, producing N-(4-(benzo[d]thiazol-2-yl)phenyl)-2- chloroacetamide, **2**. The resulted precipitate of **2** was filtered, washed, and dried in air following stirring for 2 h at room temperature. Yield: 88%; mp: 223–224 °C. ESI-MS: calcd for m/z [M-H]^+^, 302; found 302. ^1^H NMR (d6-DMSO, ppm): δ 4.36 (s, 2H), 7.46 (s, 1H), 7.52 (q, 2H, *J* 6.6 Hz), 7.86 (d, 2H, *J* 6.8 Hz), 7.96 (d, 2H, *J* 6.2 Hz), 8.02 (d, 1H, *J* 6.4 Hz), 8.14 (d, 1H, *J* 6.2 Hz). **2** (10 mmol) and di-(2-picolyl)amine (DPA, 10 mmol) were dissolved in anhydrous MeCN (80 mL). The resulted MeCN solution was heated to reflux for 24 h following addition of K_2_CO_3_ (10 mmol) and KI (0.20 g). Following filtration, the solvent was removed and the resulted residue was purified by silica gel column chromatography using CH_2_Cl_2_/AcOEt (1:5) to yield a white solid **FC-11** (39%, mp: 131–132 °C). ESI-MS: calcd for m/z [M-H]^+^, 465; found 465. ^1^H NMR (CDCl_3_, ppm): δ 8.64 (d, 2H, *J* 4.3 Hz), 8.07 (dd, 3H, *J* 14.8 and 8.33 Hz), 7.95 (d, 2H, *J* 8.6 Hz), 7.88 (d, 1H, *J* 7.9 Hz), 7.62 (dd, 2H, *J* 10.7 and 4.6 Hz), 7.48 (t, 1H, *J* 7.7 Hz), 7.36 (t, 1H, *J* 7.6 Hz), 7.32–7.26 (m, 3H), 7.20 (dd, 2H, *J* 7.4 and 4.9 Hz), 3.95 (s, 4H), 3.51 (s, 2H). **FC-11-1** was synthesized and characterized according to the procedure reported^54^. A solution of **FC-11** (0.20 g) and CuCl_2_ (0.09 g) in 20 mL methanol was filtered following stirring and heating for 2 h. Green crystals (Yield: 61%) were generated from the solution after resting for 1 week at room temperature.

A crystal of **[Cu(FC-11)Cl]**^+^ suitable for X-ray diffraction was sealed in a thin-walled quartz capillariy and mounted on a Bruker AXS Smart 1000 CCD Diffractometer equipped with graphite-monochromated Mo-Ka radiation (λ = 0.71073 Ǻ) at 298 K. The structure was resolved by direct methods and multi-scan absorption corrections were applied using the SAINT+ program. The final refinement was performed with SHELXL-97 by full-matrix least-squares methods on *F*^2^ with anisotropic thermal parameters for non-hydrogen atoms. All non-hydrogen atoms were refined anisotropically to convergence. All hydrogen atoms were added in the theoretically calculated positions and refined isotropically with fixed thermal factors (U_iso_(H) = 1.2 U_eq_ (aromatic, methylene C and imine N atoms), U_iso_(H) = U_eq_ (methyl C)). The disordered solvent molecules were treated with the program Squeeze/Platon, and their distributions were subtracted.

### Determination of stability constants

Potentiometric titration performed with an 877 Titrino plus with a 6.0262.100 glass electrode calibrated against standard buffers (Methrohm) was employed for the determination of stability constants of **FC-11** and **FC-11-1** complexes with Cu^2+^. The water-jacketed titration vessel was maintained at 25.0 °C. The titration of chelators and equimolar amount of Cu(NO_3_)_2_ was performed with small aliquots of 86.5 mM CO_2_-free NaOH solution (ionic strength, 0.1 M). The data were the averages of the results obtained by at least three independent measurements. Data analysis was carried out with the program HyperQuad program (Protonic Software, UK) using a log*K*_w_ value of 13.80^66^. Species distribution plots and titration simulations were built with the program HySS2009^67^.

### Aβ_42_ peptide experiments

The commercial Aβ_42_ peptide (GL Biochem Ltd, purifed by HPLC and characterized by ESI-MS) was dissolved in anhydrous DMSO as a stock solution (1.0 mM) and stored at −30 °C. The Cu^2+^-free Aβ_42_ aggregates were generated by diluting the stock solution into pH 7.4, 100 mM Tris-HCl or 10 mM potassium phosphate buffer (150 mM KCl, final DMSO = 1%, v/v), and incubated for 48 h at 37 °C with continuous agitation. For the Cu^2+^-bound aggregates, CuSO_4_∙5H_2_O was added at given molar ratios of Aβ_42_/Cu^2+^ before the initiation of the aggregation conditions. The concentrations of aggregates were indicated by the concentrations of Aβ_42_ peptides in all tests. The Aβ_42_/Cu^2+^ stoichiometry in the Cu^2+^-bound aggregates provided by centrifugation was determined by the inductivity coupled plasma-atomic emission spectroscopy analysis of Cu^2+^ content in the deposits and by the soluble peptide analysis in the supernatants.

### UV light exposure

The preformed Cu^2+^-free and -bound Aβ_42_ aggregates were treated for varied periods by **FC-11** or **FC-11-1** at different concentrations, or exposed to the controlled visible and UV light (deuterium UV lamp, ≤400 nm, 20 w, 750, 1500 and 3000 Lux) for varied periods, or treated by both for varied periods in pH 7.4 buffer at 37 °C. The samples were treated in darkness under the same conditions for comparison.

### Fluorescence measurements

In order to observe **FC-11** binding and Cu^2+^ sequestration of the preformed Cu^2+^-bound Aβ_42_ aggregates, fluorescence spectra (excitation at 330 nm) were recorded on a Varian Cary Eclipse spectrofluorimeter under the conditions tested. The preformed Cu^2+^-bound aggregates were treated for 0–4 h at 37 °C with **FC-11** at given ratios of [Aβ_42_]/[**FC-11**] with constant agitation prior to measurements. At least, three parallel tests were performed for all measurements.

### BCA assays

The preformed Cu^2+^-free and -bound Aβ_42_ aggregates were treated with **FC-11** or **FC-11-1** in darkness, or exposed to UV light, or treated by both for 0–4 h in pH 7.4 buffer at 37 °C. Following this treatment, the supernatants were collected by centrifugation for 20 min at 14,000 rpm. The absorbance was measured at 562 nm for all the supernatants with a Bioteck synergy-2 plate reader. The freshly prepared Aβ_42_ solution was used as control. The soluble Aβ_42_ peptide content in the supernatants was assayed by a BCA protein assay kit using bovine serum albumin as standard.

### SDS-PAGE experiments

The supernatants provided by **FC-11** or **FC-11-1** treatment of the preformed Cu^2+^-free and -bound Aβ_42_ aggregates in darkness, or by exposure to UV light, or by both, under the conditions tested were collected as above. 5 μL 5× SDS loading buffer containing 2% DMSO (v/v) was added into the supernatants. Samples were loaded, with boiling, onto 10–18% Tris-tricine gels (Biorad). Electrophoresis was performed at 100 V and gels were developed with silver or Coomassie bright blue 250.

### TEM observations

The preformed Cu^2+^-free and -bound Aβ_42_ aggregates, before and after **FC-11** or **FC-11-1** treatment in darkness, or exposure to UV light, or both under the tested conditions, were added to glow-discharged, formar/carbon 300-mesh copper grids (Electron Microscopy Sciences, Hatfield, PA) and remained for 1.5 min at room temperature. The excess sample was removed with filter paper and each grid was washed twice with ddH_2_O. Uranyl acetate (1%, 3 μL) was added to each grid and incubated for 1 min. The excess uranyl acetate was removed and grids were then dried for 15 min. Samples were visualized with a Tecnai G^2^ TEM at 200 kV.

### CD spectra

To obtain CD spectra, the preformed Cu^2+^-free and -bound Aβ_42_ aggregates were suspended, and the suspended aggregates were treated with **FC-11** or **FC-11-1** in darkness, or exposed to UV light, or treated with both, for 4 h in pH 7.4 phosphate buffer at 37 °C. CD spectra were acquired in the range of 190–260 nm with a Chirascan spectropolarimeter (Applied Photophysics, UK). The spectra were shown as an average of 5 baseline-corrected from which the buffer plus Cu^2+^ spectra were subtracted. All measurements were carried out using a 1 mm cuvette at 25 °C.

### EPR examinations

The preformed Cu^2+^-free and -bound Aβ_42_ aggregates were divided into 2 groups in pH 7.4 phosphate buffer solution containing 1% DMSO (v/v) for EPR experiments. (1) 10 μM Cu^2+^-free and -bound Aβ_42_ aggregates were incubated in darkness or exposed to 1500 Lux UV light for 4 h, respectively, in presence and absence of **FC-11** or **FC-11-1** (Aβ_42_/**FC-11** or **FC-11-1** = 1:1, 1:2). (2) As control, the phosphate buffer solutions containing 10 μM cupric sulfate, 10 μM **FC-11** or **FC-11-1**, and 10 μM **FC-11-**Cu^2+^ or **FC-11-1-**Cu^2+^ complexes, respectively, were incubated in darkness or exposed to 1500 Lux UV light for 4 h. After adding and mixing the spin trap 5,5-dimethyl-1- pyrroline-1-oxide (DMPO, 5 μL, 250 mM) and FeSO_4_ (4 μL, 0.1 mM), samples were immediately examined by a Bruker-A200 X-Band EPR spectrometer.

### Cytotoxicity assays

Cytotoxicity of the preformed Cu^2+^-free and -bound Aβ_42_ aggregates, before and after incubated with **FC-11** or **FC-11-1** in darkness, or exposed to UV light, or treated with both under the conditions tested, was estimated by 3-(4, 5-dimethylthiazol-2-yl)- 2,5-diphenyltetra-zolium bormide (MTT, Promega kit) assays^68^. Absorbance of formazan generated by MTT was determined at 590 nm with a Biotek Synergy^TM^ 2 Multi-detection Microplate Reader. SH-SY5Y human neuroblastoma and HeLa cell lines for MTT assays were obtained from China Center of Typical Culture Collection. SH-SY5Y cells were cultured in MEM containing 10% heat-inactivated fetal bovine serum (FBS, Gibco), penicillin (100 IU/mL) and streptomycin (100 μg/mL, Boster) in a 5% CO humidified environment at 37 °C. Cells were plated at a density of 10 000 cells per well on 96-well and incubated for 24 h in 90 μL fresh medium. After chelator treatment or UV exposure or both for 4 h, the aggregates for MTT assays were added, and cells were further incubated for 24 h at 37 °C. All MTT assays were conducted in room light. HeLa cells were treated and their viability was determined, as described here.

## Additional Information

**How to cite this article**: Dong, X. *et al.* Ultraviolet light triggers the conversion of Cu2^+^-bound Aβ_42_ aggregates into cytotoxic species in a copper chelation-independent manner. *Sci. Rep.* **5**, 13897; doi: 10.1038/srep13897 (2015).

## Supplementary Material

Supplementary Information
