# Supplementary material for: Ultraviolet light triggers the conversion of Cu2+-bound Aβ42 aggregates into cytotoxic species in a copper chelation-independent manner
Source: Sci Rep. 2015 Sep 9;5:13897. doi: 10.1038/srep13897 (PMC4563556; doi:10.1038/srep13897)
Supplement: Supplementary Information [file srep13897-s1.pdf]

Ultraviolet light triggers the conversion of Cu^{2+}-bound $A\beta_{42}$ aggregates into cytotoxic species in a copper chelation-independent manner

Xiongwei Dong[1,3], Zhe Zhang[1,3], Dan Zhao[1], Yaojing Liu[1], Yan Meng[1], Yong Zhang[2], Dan Zhang[1], Changlin Liu[1]

[1]Key Laboratory of Pesticide & Chemical Biology, Ministry of Education, and School of Chemistry, Central China Normal University, Wuhan (430079), Hubei; [2]School of Chemical and Materials Engineering, Hubei Polytechnic University, Huangshi (435003), Hubei; China. [3]These authors equally contribute to this work. Correspondence and requests for materials should be addressed to C. L. (liuchl@mail.ccnu.edu.cn)

Supplementary information

Figure [S1]. Fluorescence property of FC-11. (**a**) Fluorescence spectra of 10 μM **FC-11** in different organic solvents. (**b**) pH dependence of the maximum emission of 10 μM **FC-11**. (**c**) Fluorescence spectra of 10 μM **FC-11** in the presence of different metal ions (10 μM) in pH 7.4 buffer. (**d**) Fluorescence intensity at 390 nm of 10 μM **FC-11** in the presence of different metal ions (10 μM) in pH 7.4 buffer. The fluorescence intensity at 390 nm of 10 μM **FC-11** in the absence of metal ions is indicated by “Ctrl”.

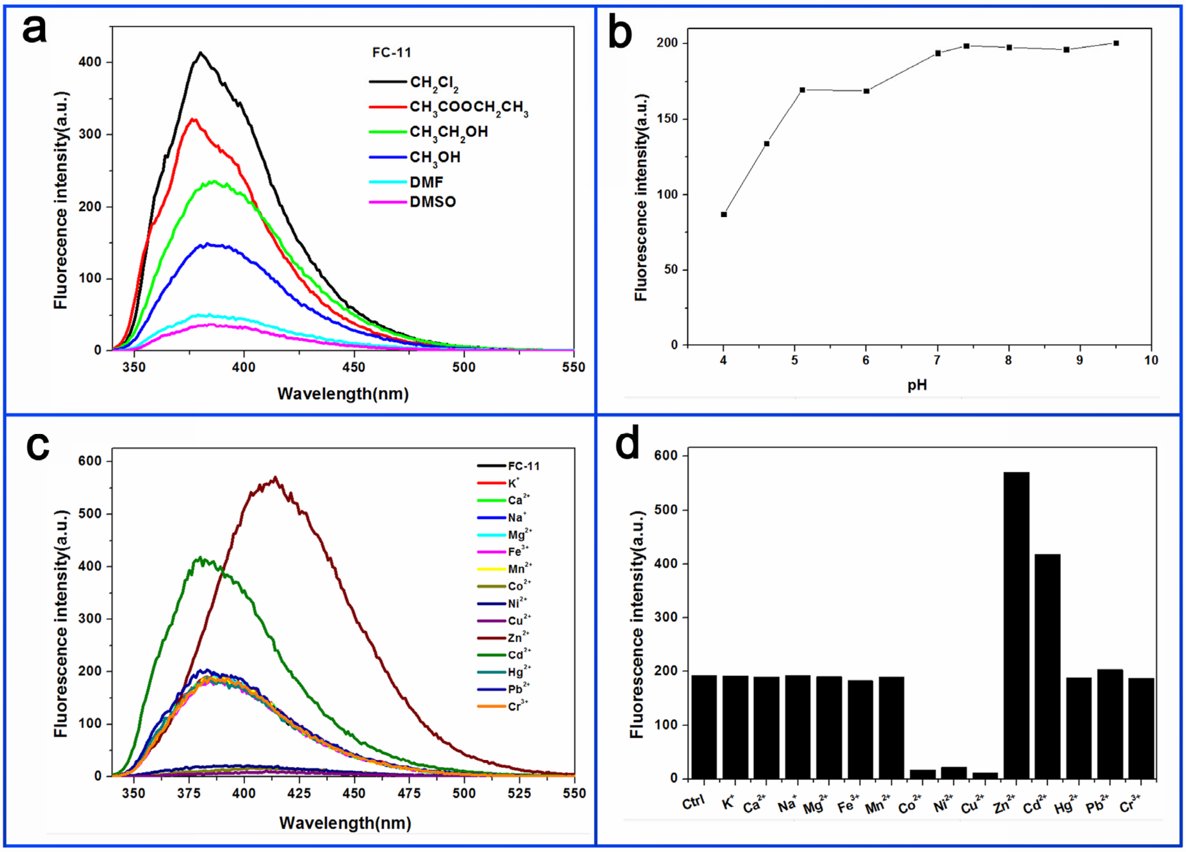

Figure [S2]. Fluorescence titration of FC-11 with cupric sulfate. (**a**) Fluorescence spectra of **FC-11** at varied ratios of Cu^{2+}/**FC-11** in pH 7.4 buffer at room temperature. (**b**) Integrated (black dot) and normalized (solid line) fluorescence intensity of 10 µM **FC-11** at 390 nm as a function of added Cu^{2+}.

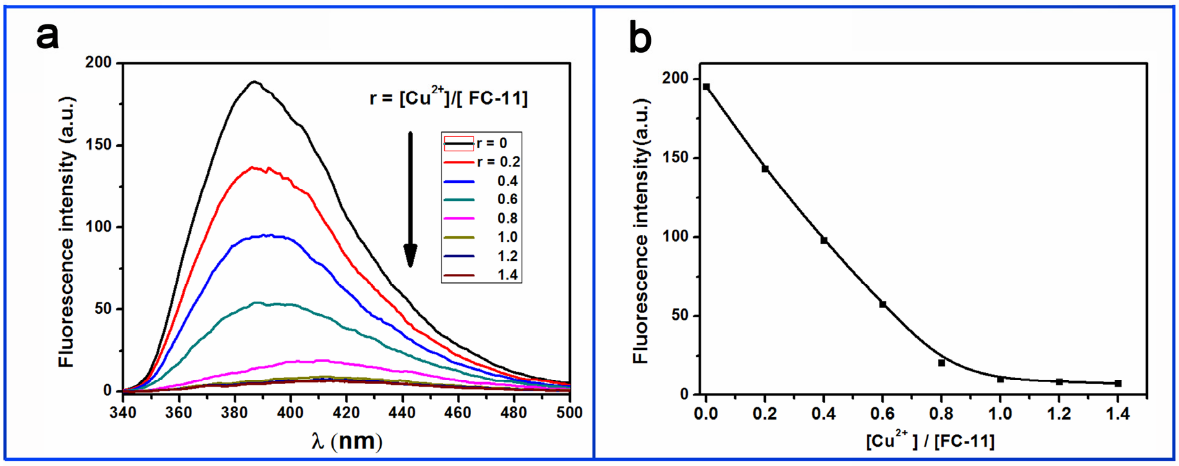

Figure [S3]. Potentiometric titrations of FC-11 or FC-11-1 with $Cu(NO_3)_2$. (a, b) Potentiometric titration curves of the solutions containing **FC-11** (**a**) or **FC-11-1** (**c**) and equimolar amount $Cu(NO_3)_2$ at 25 ℃. (**c, d**) Species distribution plots in Cu^{2+}-**FC-11** (**c**) and Cu^{2+}-**FC-11-1** systems (**d**).

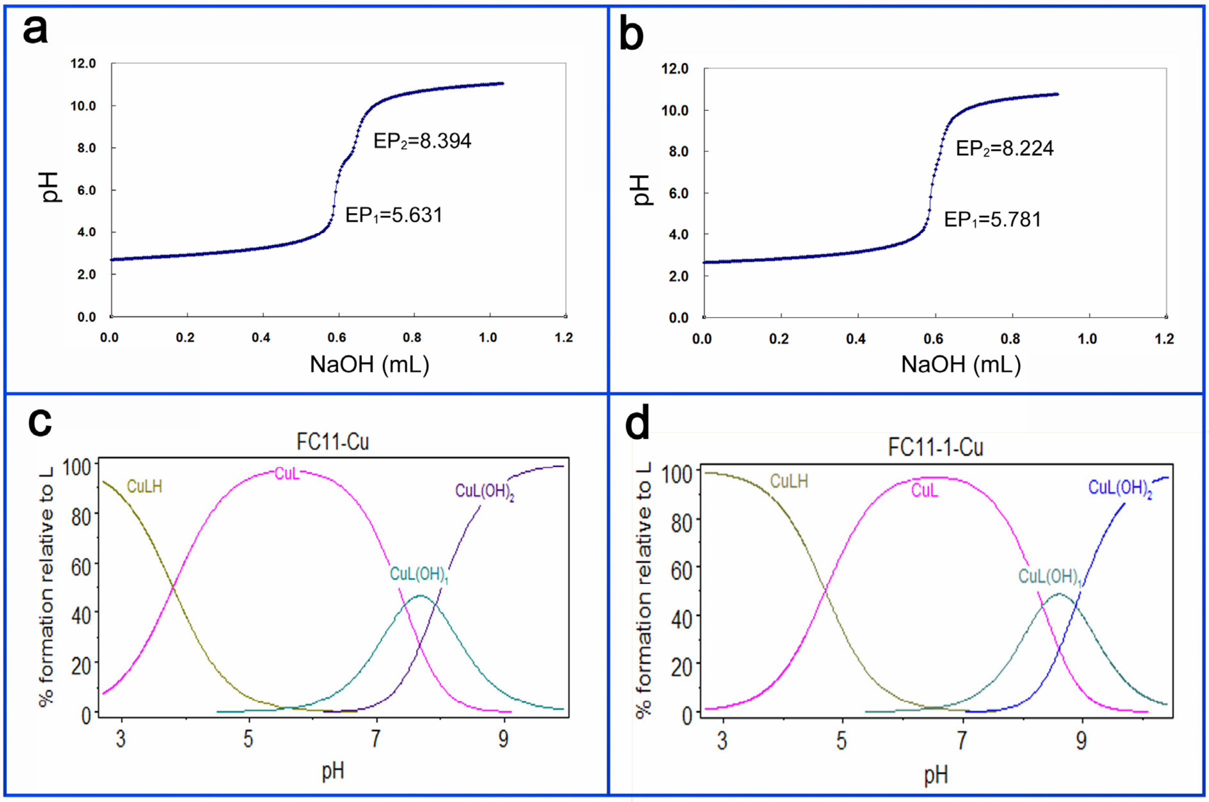

Figure [S4]. Fluorescence measurements for interactions of FC-11 or FC-11-1 with Aβ$_{42}$ aggregates. (**a**) Fluorescence intensity at 390 nm of 10 μM **FC-11** incubated for 4 h with 10 μM Cu^{2+}, 10 μM preformed Cu^{2+}-bound Aβ$_{42}$ aggregates (1:1 for Cu^{2+}/Aβ$_{42}$) at 37 ℃ and pH 7.4, respectively. (**b**) 10 μM preformed Cu^{2+}-free and -bound Aβ$_{42}$ aggregates (1:1 for Cu^{2+}/Aβ$_{42}$) were incubated for 4 h first with 10 μM **FC-11** or **FC-11-1** at 37 ℃ and pH 7.4, and then 5 μM ThT was added into these mixture solutions. Fluorescence intensity at 485 nm of ThT was measured after incubation for 5 min under the conditions tested. (**c**) Fluorescence intensity at 390 nm of 10 μM **FC-11** is linearly changed with concentrations of $CuSO_4$.

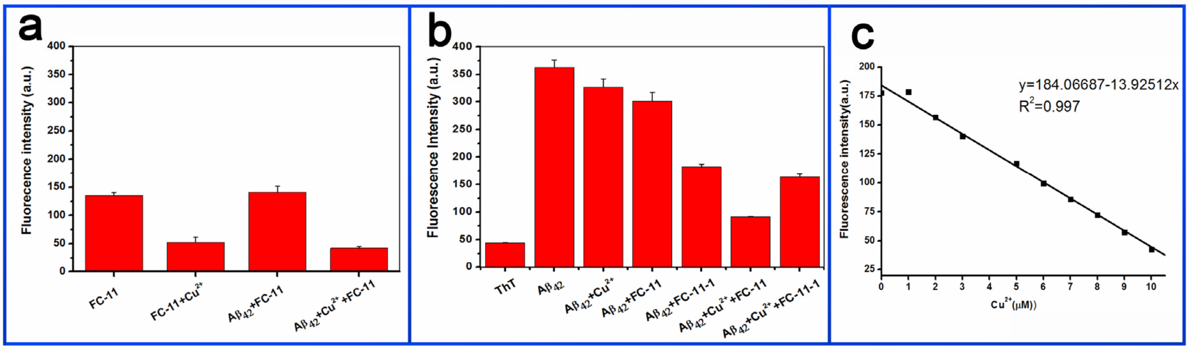

Figure [S5]. **Analysis for the dissociation of Cu^{2+}-free and -bound $A\beta_{42}$ aggregates**. (**a**) SDS-PAGE electrophoresis of 10 μM Cu^{2+}-free and -bound $A\beta_{42}$ aggregates ($Cu^{2+}/A\beta_{42}$ = 1:1) incubated in darkness or exposed to 750, 1500 and 3000 Lux UV light for 4 h at pH 7.4 and 37 ℃. (**b**) SDS-PAGE electrophoresis of 10 μM Cu^{2+}-bound $A\beta_{42}$ aggregates ($Cu^{2+}/A\beta_{42}$ = 1:1, +; 2:1, ++) incubated in darkness or exposed to 1500 Lux UV light for 4 h at pH 7.4 and 37 ℃. (**c**) SDS-PAGE electrophoresis of 10 μM Cu^{2+}-bound $A\beta_{42}$ aggregates ($Cu^{2+}/A\beta_{42}$ =1:1) treated with 1 (+) and 2 (++) equivalent **FC-11** or **FC-11-1** of $A\beta_{42}$ for 4 h in darkness or under the conditions exposed to 1500 Lux UV light at pH 7.4 and 37 ℃.

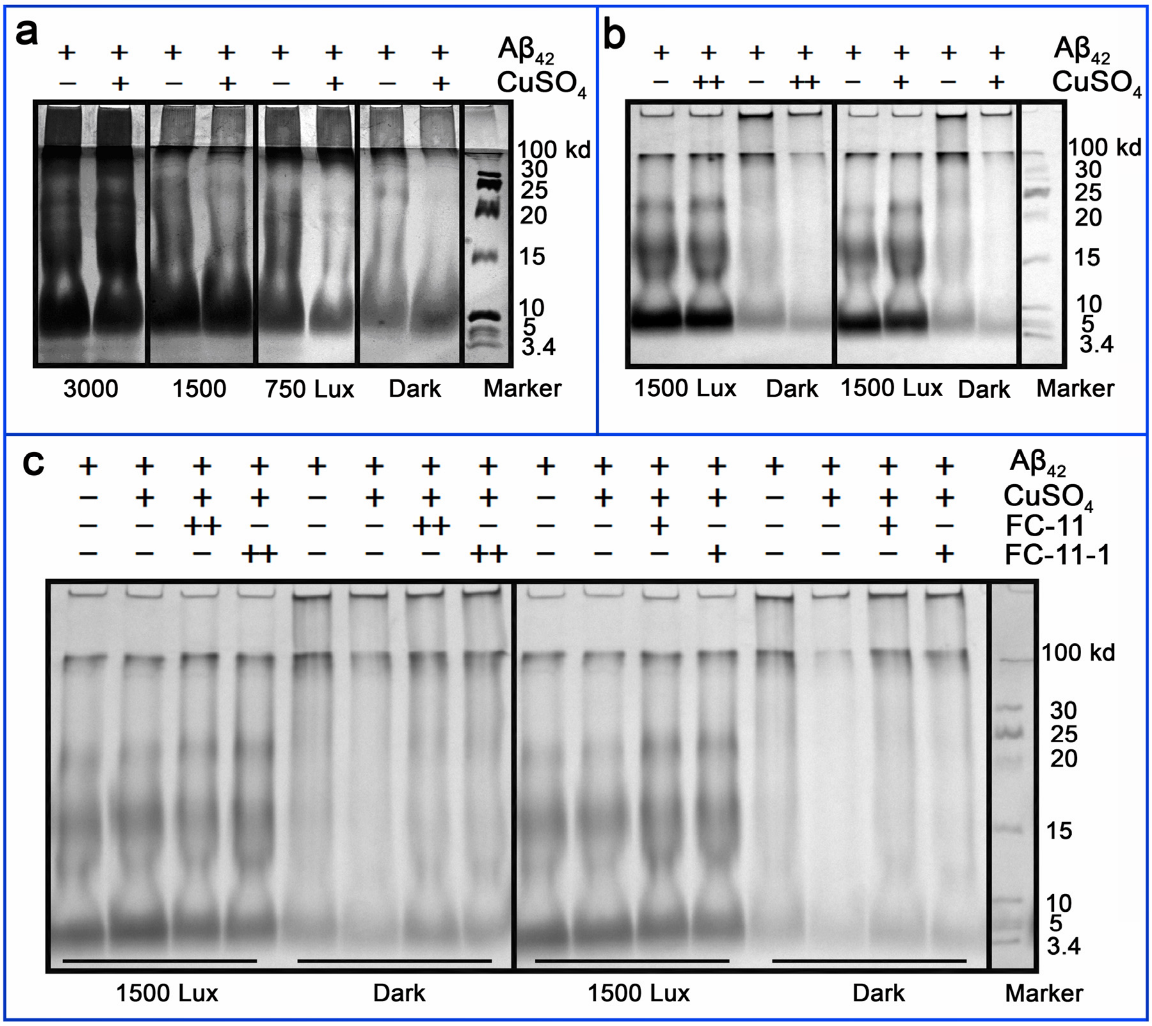

Figure [S6]. DMPO spin trap EPR detection of H_2O_2 generation. (**a**, **b**) Spin trap EPR spectra of Cu^{2+}-free Aβ_{42} aggregates in the absence of chelators following incubation in darkness (**a**) or exposure to 1500 Lux UV light (**b**) for 4 h. (**c, d**) Spin trap EPR spectra of Cu^{2+}-bound Aβ_{42} aggregates in the presence of **FC-11-1** following incubation in darkness (**c**) or exposure to 1500 Lux UV light (**d**) for 4 h.

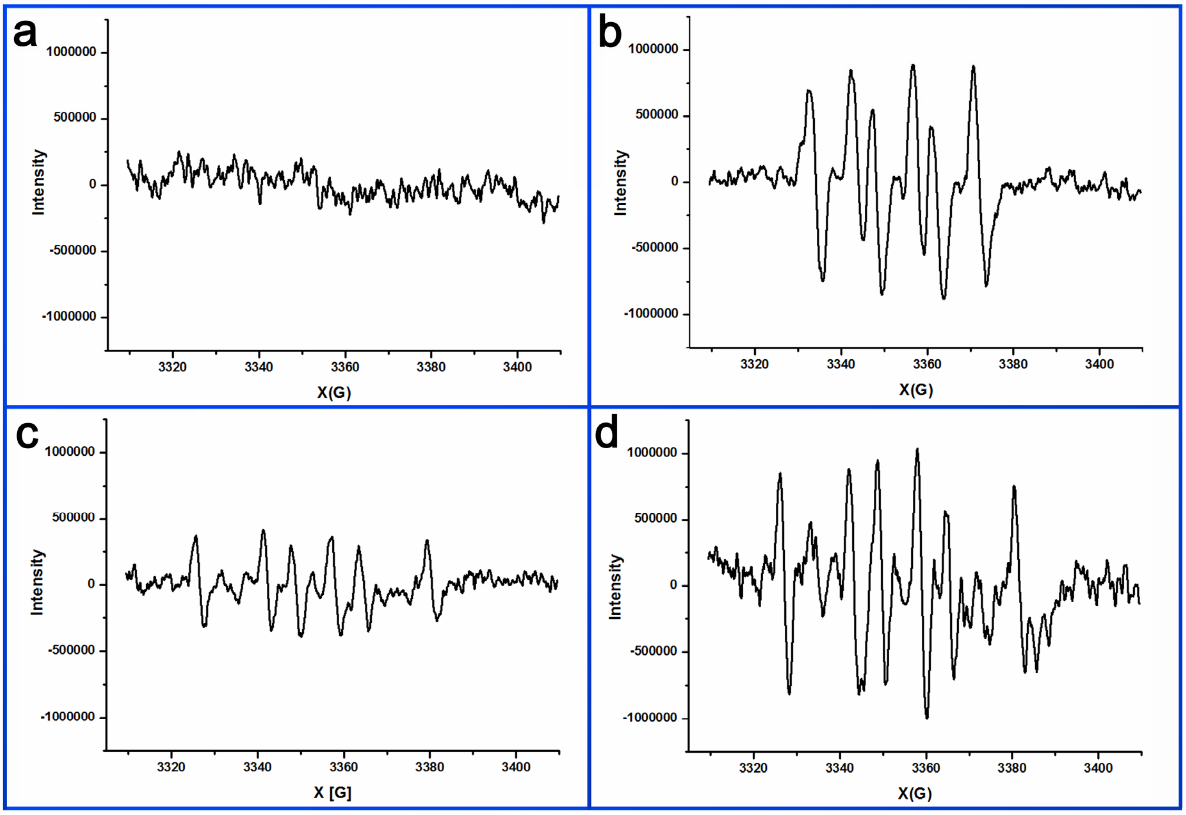

Table S1. Crystal data and structure refinement for [Cu(FC-11)Cl]$^{+}$

Empirical formula	C82 H73 Cl6 Cu3 N15 O4 S3	
Formula weight	1832.05	
Temperature	298(2) K	
Wavelength	0.71073 Å	
Crystal system	Triclinic	
Space group	P1	
Unit cell dimensions	a = 8.0958(14) Å	a= 98.980(3)°.
	b = 18.525(3) Å	b= 95.038(3)°.
	c = 35.452(6) Å	g = 94.351(3)°.
Volume	5209.7(15) Å^{3}	
Z	2	
Density (calculated)	1.168 Mg/m^{3}	
Absorption coefficient	0.868 mm^{-1}	
F(000)	1878	
Crystal size	0.20 x 0.20 x 0.10 mm^{3}	
Theta range for data collection	0.58 to 25.01°.	
Index ranges	-9<=h<=9, -22<=k<=21, 0<=l<=42	
Reflections collected	18275	
Independent reflections	18275 [R(int) = 0.0000]	
Completeness to theta = 25.01°	99.4 %	
Absorption correction	None	
Max. and min. transmission	0.9182 and 0.8455	
Refinement method	Full-matrix least-squares on F^{2}	
Data / restraints / parameters	18275 / 0 / 1042	
Goodness-of-fit on F^{2}	0.913	
Final R indices [I>2sigma(I)]	R1 = 0.0672, wR2 = 0.1810	
R indices (all data)	R1 = 0.1023, wR2 = 0.1983	
Largest diff. peak and hole	0.833 and -0.554 e.Å^{-3}	
